# Knowledge, attitude and practice towards anthrax in northern Ethiopia: a mixed approach study

**DOI:** 10.1186/s12879-020-05544-z

**Published:** 2020-11-10

**Authors:** Gebremedhin Romha, Weldemelak Girmay

**Affiliations:** Department of Veterinary Public Health and Food Safety, College of Veterinary Sciences, Mekelle University, Mekelle, Ethiopia

**Keywords:** Anthrax, Ethiopia, Knowledge-attitude-practice, Tigray

## Abstract

**Background:**

Anthrax is the second most highly prioritized zoonotic disease in Ethiopia due to its negative impact at the household level, causing disease and production losses in livestock and severe disease in humans. This study seeks to assess the knowledge of, attitudes towards, and practices addressing (KAPs) anthrax in the communities of Eastern Tigray, Northern Ethiopia.

**Methods:**

A cross-sectional survey was conducted concurrently with focus group discussions (FGDs) and key informant interviews (KIIs) between May 2019 and April 2020. A total of 862 respondents participated in the questionnaire survey. Of these, 800 were local community members while 62 were professionals working at health service institutions. In addition, qualitative data were collected using six FGDs and 11 KIIs.

**Results:**

Sixty-two percent (496/800) of the community respondents said that they were aware of anthrax while 38% (304/800) of them did not. Only 9.3% (74/800) of the respondents reported that the causative agent of anthrax is germs/microbial. About 56.5% (35/62) of professional respondents said that it is bacterial. More than 60% (64.1%, 513/800) of the respondents did not know that whether the disease was zoonotic or not. Regarding clinical signs, 26.3 (210/800) and 36.8% (294/800) of the respondents could identify at least one in animals and humans, respectively, while 21.3 (170/800) and 20.1% (161/800) knew one or more transmission routes in animals and humans, respectively. Moreover, 43.4% (347/800) and 45.6% (365/800) of the respondents mentioned one or more control/prevention method(s) in animals and humans, respectively. Regarding qualitative results, some of the participants knew the disease (in animals) by their local names: *Lalish* and *Tafia* (splenomegaly), and *Gulbus* (abdominal cramps and shivering). Some reported that anthrax was exclusively a human disease while others recognized its zoonotic potential after the clinical signs in both animals and humans were listed.

**Conclusion:**

The KAP of the participants regarding anthrax was low. There was no consistent understanding of the disease among the participants. The study also revealed that the participants did not receive consistent, adequate, and continuous education regarding the disease.

**Supplementary Information:**

The online version contains supplementary material available at 10.1186/s12879-020-05544-z.

## Background

Anthrax is a neglected tropical zoonotic disease of economic and public health importance [1]. It is estimated that 20,000–100,000 incidents of human anthrax occur per year globally [2] with a significant number of cases in Chad, Ethiopia, Zambia, Zimbabwe and India [1]. However, the true disease burden is likely unknown, as poor surveillance systems and unreliable reporting are prevalent [3]. The causative agent of anthrax is *Bacillus anthracis* (*B. anthracis*), which primarily infects herbivores and secondarily humans [4, 5]. Pasture contaminated with anthrax spores is the most common source of infection for ruminants [6–8]. Animals may also become infected through concentrated feed [9].

The occurrence of anthrax outbreaks in a particular location mostly depends on multiple factors, which include unique characteristics of the bacterium, environmentally related features, animal densities and human activities [10, 11]. Anthrax outbreaks have been associated with ecological, demographic, and sociocultural factors [12, 13]. The occurrence of human cases is often highly correlated with animal anthrax outbreaks [14]. Anthrax epidemics are frequent in the dry season and are often associated with the onset of the first rains [15, 16]. During the dry season, the grass is short and animals are forced to graze closer to the ground, increasing the opportunity to ingest anthrax spores [17], especially when anthrax-infected carcasses and butchering waste have been disposed of in environments where ruminants live and graze [18]. Spores may also be spread in the environment through scavenging birds, animals, and water [6, 19]. Repeated anthrax outbreaks in animals without vaccination have reported [18]; however, ongoing vaccination programs can break the cycle of transmission in domestic animals [7, 18].

Human infection is often associated with eating the meat of infected animals [20, 21], and as a result of coming into contact with infected animals or contaminated animal materials during agricultural activities, including the butchering of livestock or industrial exposures through the processing of hair and bone [5, 20–24]. One of the drivers that may contribute to the persistence of anthrax is human behavior [25]. For instance, in Kenya, it was reported that human anthrax cases most often occur linked to animal anthrax. In most cases, human behaviors, especially slaughter and consumption of meat from animal anthrax cases, has been implicated [26]. In Zambia, popular cultural practices that involved the exchange of animals between herds has facilitated subsequent transmission of anthrax [13]. In Tanzania, it has been documented that demographic characteristics (e.g., sleeping on animal skins, contact with infected carcasses through skinning and butchering, and not having formal education) were linked to exposure for anthrax infection [20]. Because animals are an important asset to the communities affected, the death of an animal may result in the consumption of infected meat and the use of animal products, potentially leading to infections. In Bangladesh, it was identified that hides have been skinned and sold from dead and discarded carcasses [18]. This is exacerbated by the fact that a family may consume and sell some of the meat in order to salvage some losses from the death of the animal [17, 18, 25, 27].

According to Pieracci et al. [28], the United States Centers of Disease Control (US-CDC) and other concerned Ethiopian and North American organizations have prioritized five zoonotic diseases in Ethiopia based on 1) severity of disease in humans, 2) proportion of human disease attributed to animal exposure, 3) burden of animal disease, 4) availability of interventions and 5) existing inter-sectoral collaboration. According to this list, anthrax ranks second based on its negative impacts at the household level due to disease and production losses in livestock and severe disease in humans in the country. In Ethiopia, government reports indicate that a total of 5197 and 26,737 cases and 86 and 8523 deaths of human and animal anthrax, respectively, were documented from 2009 to 2013 [29]. It should be noted that deficiencies in diagnostic testing services and the non-specific presentation of many zoonoses suggest under-diagnosis and failure to reinforce awareness creation [30]. One study reported that the lack of awareness of zoonotic diseases was due to poor communication between veterinarian and human health-care professionals and the lack of involvement of educated family members in farming activities [31]. Specifically, in Zambia, it has been reported that the persistence of anthrax outbreaks was linked to perceptions, beliefs and practices of farmers; for example, cattle farmers are reluctant to have their livestock vaccinated against anthrax because of a perceived low efficacy of the vaccine. In addition, farmers do not trust professional staff and their technical interventions [13].

One sensible control strategy involves establishing a bond of trust between responsible authorities and those who have had a positive impact on communities through effective communication [32]. This might contribute to improved compliance regarding control measures and the application of evidence-based interventions (both technical and locally acceptable) [27]. It has been acknowledged that cultural issues are always an important component of health, especially in agrarian communities [33]. Human behavior [25] and socio-demographic factors [20] could affect the KAP of a given community towards anthrax. Demonstrating evidence of KAP regarding anthrax can be used to determine the prevention strategy for the disease. For these reasons, this study aimed at assessing the knowledge of, attitude towards, and practices addressing anthrax at Adigrat town, Ganta Afeshum and Gulomkada districts.

## Methods

### Description of study area

**Fig. 1:**
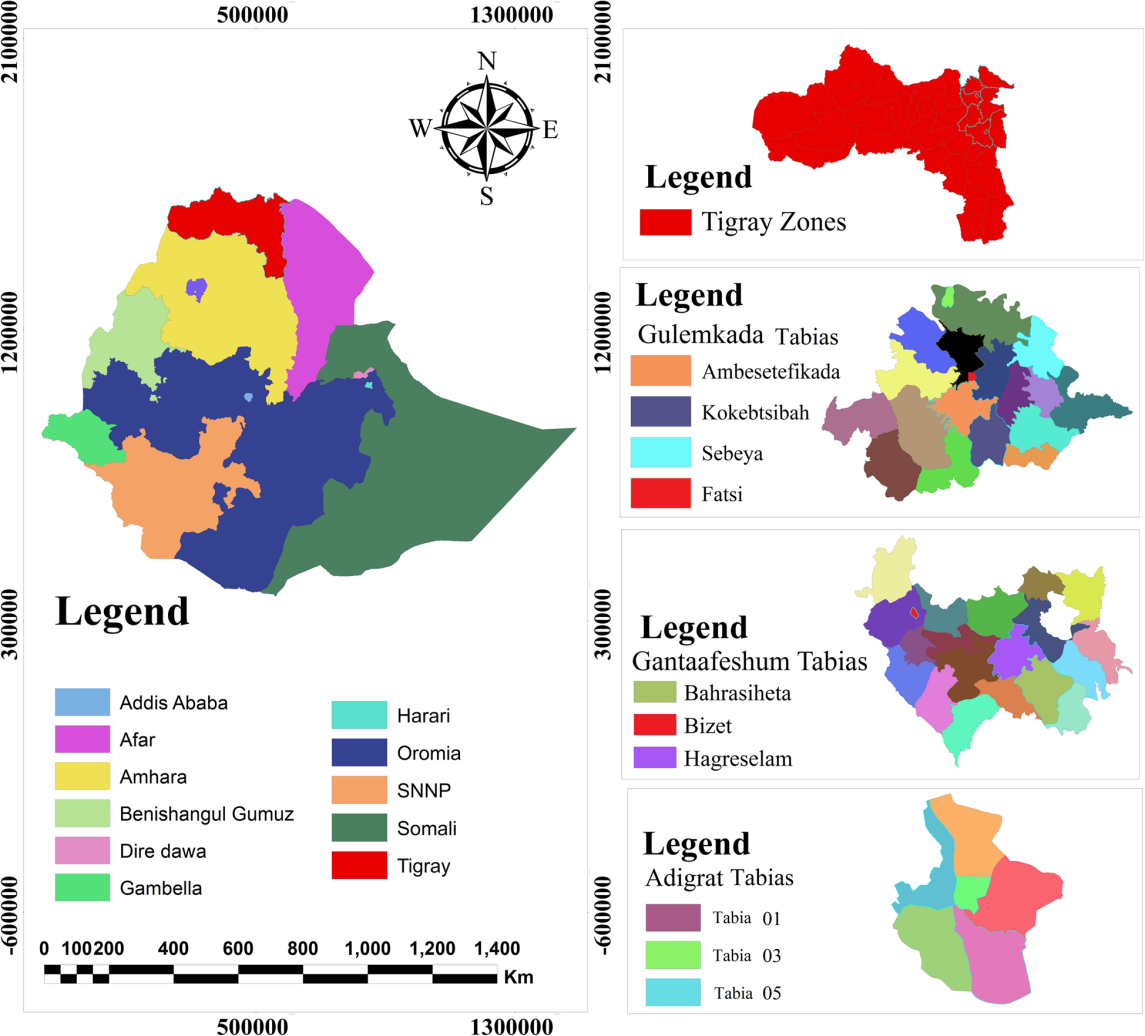
Map of the study area. Source: Ethiopian Statistical Agency, EthioGIS mapserver Ethiopia, it is freely available at https://www.ethiogis-mapserver.org. But the study area was produced from Ethio-GIS using Arc GIS 10.3 software

The eastern zone is one of the seven rural zonal administrative units of Tigray Regional State which consists of nine districts (two are town districts while the remaining seven are rural districts). Adigrat is the capital town of the eastern zone. The zone is located at 14^0^16^′^N 39^0^27^′^E longitude and 14^0^16^′^N 39^0^27^′^E latitude; the altitude ranges from 2000 to 3000 m above sea level. The average annual rainfall is 552 mm and the average temperature is 16 °C. The study was conducted in three districts of the Eastern Zone, namely, Adigrat town, Ganta-afeshum, and Gulomkada (Fig. 1).

### Definitions

1. A zone is a political administrative unit, larger than district but smaller than regional state.
2. Ketena and Kushet (urban and rural, respectively) are the smallest administrative units in the study region (Tigray). Both are synonymous with a parish.
3. A tabia is an administrative unit larger than a ketena/kushet but smaller than a district.
4. Community member: in this paper, community member means respondents who participated in the questionnaire survey; it is only used to differentiate respondents who are not professional/experts, FGD participants and key informants.

### Study design

The study used a mixed-method design employing both quantitative and qualitative methods. A cross-sectional survey was concurrently conducted with FGD and KII from May 2019 to April 2020.

### Healthcare sector description

Animal and human healthcare sectors were identified before the actual data collection had begun. There were nine veterinary clinics in the study districts. Ganta-afeshum and Gulomkada had four clinics for each while Adigrat town had one veterinary clinc. They provided veterinary service based on clinical signs with a few broad-spectrum medications. Most of the rural tabias had no permanent veterinary facilities. They were served by the nearest clinic for vaccination and sometimes for case management. Healthcare coverage is better in the human than the animal sector. Formal clinical services were provided through health posts, clinics/health centers and hospitals. Our rural study tabias had one health center each except Bahrasehita and Anbesetefikada which received healthcare services from neighboring tabias. Fatsi (a tabia found in Gulomakda district) had one primary hospital. Adigrat town district had one general hospital and two health centers.

### Sample size determination and sample selection

Sample size for the community members was determined using the single population formula: $=\frac{z^2 pq}{e^2}$ . Since the prevalence of anthrax was not detected previously in the area, n = $\frac{(1.96)^2\ (0.5)(0.5)}{(0.05)^2}$ = 384. With a 95% CI (z = 1.96), a 5% level of precision, a design effect of two and a 10% non-response rate, the total *n* = 806 (six questionnaires were droped due to incomplete data). In addition, 62 animal and human professional respondents were included in this study. A multistage cluster sampling was performed. Districts and tabias were selected based on logistic feasibility and history of anthrax outbreak, and treated as first and second sampling units, respectively. In total, 10 tabias were included in the study. Three tabias from each district, were selected: Adigrat (01, 03 and 05 tabias), Ganta-afeshum (Bizet, Bahrasehita and Hagereselam) and Gulomkada (Fatsi, Kokobtsibah, Anbesetefikada). One tabia (Sebeya) from Gulomkada which had anthrax outbreak in 2018 was purposively included. All parishes under the selected tabias were included. The sample size allocated to each tabia and parish was proportional to its population size. The number of households and populations of the study tabias and parishes were obtained from the administrative offices of each tabia. Lists of households were not available. Hence, the respondents’ households were selected using systematic random sampling while all health professionals available at the time of visiting were included.

### Data collection

#### Quantitative

A structured questionnaire consisting of mostly close-ended questions was prepared. It was translated into the local language, *Tigrigna*, and then translated back into English to maintain consistency. Interviewers were selected who were both native Tigrigna speakers and had a university degree. Both males and females were selected as interviewers; all underwent training. In addition to the content of the questionnaire, interviewers underwent training on anthrax consisting of supportive pictures which showed clinical signs including cutaneous lesions in humans and bleeding from natural orifices in dead animals. The authors themselves participated in data collection. The questionnaires were pretested on 35 respondents: 30 questionnaires (prepared for the community members) were tested on urban and rural residents of the study area, and five on university instructors (questionnaire prepared for professionals). Some questions were modified following the pretest. In this study, a total of 862 respondents were interviewed. Eight hundred of whom were urban and rural dwellers. Sixty two were professionals/experts working at animal and human health service institutions: 49 human health practitioners (HHPs) and 13 animal health experts (AHEs). For the community, one person per household, at least 18 years old, was interviewed. The interview was held face to face with the interviewers. Health workers were given the questionnaire and allowed to answer the questions by themselves.

The questionnaires were of two types: one for the community members (Additional file 1: Questionnaire A) and another for professionals (Additional file 1: Questionnaire B) with slight differences between questionnaries for animal and human professionals. The first questionnaire addresses socio-demographic information of the respondent (age, sex, educational status, occupation, religion), animal ownership, questions related to knowledge (e.g. knowledge of the disease, source/cause of the disease, signs of the disease in animals and humans, transmission routes to animals and humans, prevention methods in animals and humans), attitude (e.g. seriousness of the disease in animals and humans and the importance of vaccination) and practice related questions (e.g. animal vaccination frequency; medication of animals and humans). The second questionnaire aimed at assessing the knowledge of the disease amongst health professionals. The majority of the questionnaire’s components were the same as the first one. However, some additional questions were incorporated: questions related to knowledge (e.g. etiology of the disease, form of anthrax, transmission route for each form), questions related to outbreak, and case admission to animal and human healthcare centers (e.g. occurrence of an outbreak in animals and humans, number of cases admitted in animal and human healthcare center, number of recoveries and deaths in animals and humans).

#### Qualitative

Six FGDs and 11 KIIs were conducted. The FGD participants were individuals who did not take part in the questionnaire survey. Each group was composed of both sexes, farmers and local leaders. The FGD meetings were moderated by the researchers and each discussion was held until it reached a saturation point, an average of 40 min. The mean age of the FGD participants was 44 (range: 24–78). The FGDs consisted of 7–15 (total of 58) participants. Focus Group Discussions were held in six tabias: three from Gulomkada (Fatsi, Anbesetefikada and Sebeya), one from Adigrat town and two from Ganta-afeshum (Hagereselam and Bizet). The FGDs always began with the animal health coordinators highlighting the purpose of the discussion. The participants were encouraged to speak their opinions. They were allowed at times to to speak amongst themselves (e.g, to help each other) in order to better explore the topic at hand until their attention was directed back to the stage by the researchers.

Eleven KIIs were held with officers responsible for anthrax control/prevention: Amongst the 11 officers interviewed, seven were human health officers (HHOs) and four were animal health officers (AHOs): five from Gulomkada (HHOs, *n* = 3; AHOs, *n* = 2), three from Ganta-afeshum (HHOs, n = 2; AHOs, *n* = 1) and three from Adigrat town (HHOs, n = 2; AHOs, n = 1). One (1/11) of them was female while the remaining interviewees (10/11) were male.

Interviews with Key Informants and Focus Group Discussions were held in the local language, *Tigrigna*. Both the FGDs and the KIIs were conducted using interview guides with open ended questions/themes (Additional file 1: Questionnaire C) to allow the participants to freely express their views and thoughts in their own words on the subject matter, and were recorded on a digital recorder after getting verbal consent from the participants. During the interview, the interviewer was deliberate in keeping the conversation on topic.

### Data management and statistical analysis

Quantitative data were run using STATA statistical software (Version 14.0, Stata Corp, College Station, Texas 77,845 USA). To ensure quality, data were crosschecked independently by the researchers. Frequency distribution tables were used to quantify the knowledge of respondents regarding anthrax, its causative agent, zoonotic nature, symptoms, transmission, and control/prevention methods concerning socio-demographic factors such as age, sex, educational level, district, occupation, religion and animal ownership. Moreover, with 95% confidence intervals, a logistic regression model was used to evaluate the association between the outcome (e.g., knowledge regarding the disease’s zoonotic nature) and the aforementioned socio-demographic variables. A *P*-value *<* 0.05 was considered statistically significant.

Qualitative data collected through an audio recorder first were saved as digital files and then were translated from the local language *Tigrigna* into English. Thematic analysis was used as described in Graneheim and Lundman [34]. The narratives were read several times to understand the whole sense of the text. After determining the major themes, texts were extracted and brought under the appropriate theme. Illustrative quotations that represented the themes were used in the results.

## Results

### Quantitative

#### Socio-demographic characteristics

Of the 800 rural and urban residents, 54.9 (439/800) and 45.1% (361/800) were males and females, respectively. The mean age of the respondents was 46.7 (95% CI: 45.6–47.9) with a range of 18–90 (median age 45) years. About 38.8 (310/800), 31.6 (253/800) and 29.6% (237/800) were from Gulomkada, Gant-afeshum and Adigrat districts, respectively. Regarding the professionals, 49.2% (30/61) were males while 50.8% (31/61) were females. More than 60% (61.7%, 37/62) were diploma graduates while 33.3 (20/62) and 5% (3/62) were respectively first and second degree holders (Table 1).

**Table 1:** Socio-demographic characteristics of the respondents

Variable		Professionals (*n = 62*)		Community (*n = 800*)	
		Number	%	Number	%
Sex	Female	31	50.8	361	45.1
	Male	30	49.1	439	54.9
Age	18–25	10	23.3	68	8.6
	26–33	22	51.2	124	15.7
	34–41	5	11.6	146	18.4
	42–49	3	7.0	116	14.7
	50–57	3	7.0	115	14.5
	58–65	0	0	101	12.8
	> 65	0	0	122	15.4
Education	No formal education	–	–	309	38.6
	1–4	–	–	110	13.8
	5–8	–	–	160	20
	9–10	–	–	136	17
	11–12	–	–	38	4.8
	Diploma	37	61.7	27	3.4
	Degree	20	33.3	18	2.3
	MSc	3	5	2	0.3
Occupation	Civil servant	–		39	4.9
	Merchant	–		145	18.1
	Farmer	–		449	56.1
	Daily worker	–		44	5.5
	Students	–		18	1.6
	Other	–		12	1.5
	Unemployed	–		98	12.3
Animal ownership	Have	13	21	452	56.5
	Have not	49	79	348	43.5
District	Adigrat	25	40.3	237	29.6
	Gulomkada	17	27.4	310	38.8
	Ganta-afeshum	20	32.3	253	31.6
Religion	Orthodox	54	94.7	770	96.7
	Catholic	2	3.5	6	0.8
	Muslim	1	1.8	19	2.4
	Protestant	–	–	1	0.1
Profession	Human health	49	79	–	–
	Veterinarian	13	21	–	–

#### Knowledge of the disease, its causative agent and zoonotic nature

##### Community members

Sixty two percent (496/800) of the respondents said that they knew of the disease anthrax, locally (in *Tigrigna*) called *Megerem*. However, 38% (304/800) of them said that they did not know of the disease. The proportion who knew of anthrax was greater in female (63.4%, 229/361) than in male (60.8%, 267/439) respondents. The majority (77.6%, 621/800) of the respondents reported that they did not know the cause of the disease (Table 2). However, of the respondents (22.4%, 179/800) who believed to have known the cause of the disease: Only 9.3% (74/800) said that the disease was caused by a microbe/germ. About 8.9% (71/800) believed that the disease was God given, and 4.3% (34/800) mentioned other entities (heredity, witchcraft, lack of sanitation, hunger, insects, and thirst) as the causative agent of the disease. Of the respondents who claimed to know the disease, the majority (63.9%, 317/496) of them responded that they did not know the causative agent of the disease (Table 2). Regarding the zoonotic importance of the disease, 64.1% (513/800) did not know whether the disease was zoonotic or not. About 20.1% (161/800) of respondents claimed that anthrax was zoonotic while 15.8% (126/800) said that the disease was not zoonotic (Table 2). The level of knowledge of respondents regarding anthrax increased with age (Additional file 1: Fig. A).

**Table 2:** Knowledge on the disease, its cause and zoonotic nature by the community member respondents

Variable		Knowledge on the disease		Knowledge on the cause of the disease				Zoonotic nature		
		Yes (n/%)	No (n/%)	Germ (n/%)	God given (n/%)	^a^Other (n/%	Don’t know (n/%)	Yes (n/%)	No (n/%)	Don’t know (n/%)
Sex (*n = 800*)	Female	229/63.4	132/36.6	22/6.1	37/10.3	14/3.9	288/79.8	66/18.3	62/17.2	233/64.5
	Male	267/60.8	172/39.2	52/11.9	34/7.7	20/4.6	333/75.9	95/21.6	64/14.6	280/63.8
	Total	496/62	304/38	74/9.3	71/8.9	34/4.3	621/77.6	161/20.1	126/15.8	513/64.1
Age (*n = 792*)	18–25	23/33.8	45/66.2	3/4.4	4/5.9	1/1.5	60/ 88.2	12/17.7	5/7.4	51/75
	26–33	59/47.6	65/52.4	11/8.9	4/3.2	1/0.8	108/87.1	20/ 16.1	9/7.3	95/76.6
	34–41	88/60.3	58/39.7	12/8.2	16/11	5/3.4	113/77.4	34/23.3	20/13.7	92/63
	42–49	77/66.4	39/33.6	17/14.7	10/8.6	5/4.3	84/72.4	28/24.1	19/ 16.4	69/59.5
	50–57	80/69.6	35/30.4	16/13.9	13/11.3	3/2.6	83/72.2	26/22.6	21/18.3	68/59.1
	58–65	80/79.2	21/ 20.8	7/6.9	7/6.9	10/9.9	77/76.2	18/17.8	28/27.7	55/54.5
	> 65	84/68.9	38/31.1	6/4.9	17/13.9	9/ 7.4	90/73.8	20/16.4	23/18.9	79/64.8
	Total	491/62	301/38	72/ 9.1	71/9	34/ 4.3	615/ 77.7	158/20	125/15.8	509/ 64.27
Education (*n = 800*)	No formal education	205/66.3	104/33.7	24/7.8	36/11.7	15/4.9	234/75.73	49/15.9	64/20.7	196/63.4
	1–4	78/70.9	32/29.1	13/11.8	11/10	4/3.6	82/74.6	29/26.4	12/10.9	69/62.7
	5–8	104/65	56/35	16/10	12/7.5	10/6.3	122/76.3	37/23.1	28/17.5	95/59.4
	9–10	64/47.1	72/52.9	7/5.2	9/6.6	3/2.2	11/86	17/12.5	13/9.6	106/77.9
	11–12	18/47.4	20/52.6	2/5.3	2/5.3	0	34/89.4	10/26.3	4/10.5	24/63.1
	Diploma	16/59.3	11/40.7	7/25.9	1/3.7	2/7.4	17/63	11/40.7	4/14.8	12/44.4
	1st degree & above	11/55	9/45	5/25	0	0	15/75	8/40	1/5	11/55
	Total	496/62	304/38	74/9.3	71/8.9	34/4.3	621/77.6	161/20.1	126/15.8	513/64.1
Occupation (*n = 800*)	Civil servant	28/71.8	11/28.2	9/23.1	2/5.1	6/15.4	22 56.4	15/38.5	8/20.5	16/41
	Merchant	72/49.7	73/50.3	9/6.2	14/9.7	3/2.1	119/82.1	29/20	17/11.7	99/68.3
	Farmer	288/64.1	161/35.9	46/10.2	34/7.6	17/3.8	352/78.4	78/17.4	70/15.6	301/67
	Daily worker	28/63.6	16/36.4	3/6.8	4/9.1	2/4.6	35/79.6	14/31.8	9/20.5	21/47.7
	Students	4/30.7	9/69.2	1/7.7	0	0	12/92.3	1/7.7	2/15.4	10/76.9
	^b^Other	9/75	3/25	3/25	1/8.3	0	8/66.7	6/50	1/8.3	5/41.7
	Unemployed	67/68.7	31/31.6	3/3.1	16/16.3	6/6.1	73/4.5	18/18.4	19/19.4	61/62.2
	Total	496/62	304/38	74/9.3	71/8.9	34/4.3	621/77.6	161/20.1	126/15.8	513/64.1
Animal ownership (*n = 800*)	Have	298/65.9	154/34.1	52/11.5	42/9.3	19/4.2	339/75	87/19.3	82/18.1	283/62.6
	Have not	198/56.9	150/43.1	22/6.3	29/8.3	15/4.3	282/81	74/21.3	44 (12.6)	230/66.1
	Total	496/62	304/38	74/9.3	71/8.9	34/4.3	621/77.6	161/20.1	126/15.8	513/64.1
District (*n = 800*)	Adigrat	152/64.1	85/35.9	20/8.4	24/ 10.1	15/6.3	178/75.1	69/29.1	53/22.4	115/48.5
	Gulomkada	155/50	155/50	44/14.2	8/ 2.6	9/2.9	249/80.3	53/17.1	20/6.5	237/76.5
	Ganta-afeshum	189/74.7	64/25.3	10/4	39/15.4	10/4	194/76.7	39/15.4	53/21	161/63.6
	Total	496/62	304/38	74/ 9.3	71/8.9	34/4.3	621/77.6	161/20.1	126/15.8	513/64.1

^a^Heredity, witchcraft, lack of sanitation, hunger, insects, thirst

^b^Private company worker, retired

A logistic regression model was used to determine the effect of socio-demographic factors (age, sex, district, level of education, occupation and animal ownership) on the knowledge of anthrax and its zoonotic nature. Accordingly, age, sex, district, occupation, and animal ownership had a statistically significant association with knowledge of the disease anthrax. Respondents who were 58–65 years olds were found to be seven times more knowledgeable about the disease (OR: 6.7; 95% CI: 2.80–16.01; *p* < 0.001) than those 18–25 years old. Male (OR: 0.6; 95% CI: 0.40–0.85; *p* < 0.005) and merchant (OR: 0.3; 95% CI: 0.12–0.80; *p* = 0.016) respondents, and participants from Gulomkada (OR: 0.3; 95% CI: 0.19–0.53; *p* < 0.001) had a significantly lower level of knowledge about anthrax than females, civil servants, and participants from Adigrat, respectively. Respondents who owned animals (OR: 1.8; 95% CI: 1.10–2.83; *p* = 0.02) had a better knowledge of anthrax than respondents who did not own animals. However, only districts had a statistically significant association with an awareness of the zoonotic nature of the disease. Respondents from Ganta-afeshum (OR: 0.4; 95% CI: 0.19–0.65; *p* < 0.001) had a lower level of awareness regarding the zoonotic nature of the disease than respondents from Adigrat. The respondents of the community members learned about anthrax in various ways: 55.8% (446/800) from family, friends and neighbours/colleagues, 2.8% (22/800) from health experts, 2.1% (17/800) from radio, and 2% (16/800) from veterinary experts (Additional file 1: Table A and Fig B).

##### Professionals

About 56.6% of the respondents said that the causative agent of the disease was bacterial while 33.9% of them did not know. However, 9.7% claimed that the causative agent of anthrax was another organism (e.g., Leishmania and flies). The cutaneous form (67.2%) was the most well known form of the disease. More than 90 % (90.1%) of the professional respondents knew that anthrax was zoonotic (Table 3). Socio-demographic factors such as age, sex, profession (animal and human experts), and level of education were analysed using a logistic regression model to determine whether there was a statistical association with the knowledge of the causative agent and zoonotic nature of anthrax or not. None of them was found to be statistically significant.

**Table 3:** Knowledge on the disease, its cause and zoonotic nature by the professional respondents

Variable		Knowledge on the cause of the disease			Forms of the disease^a^			Zoonotic nature		
		Bacteria (n/%)	^b^Other (n/%)	Don’t know (n/%)	Cutaneous (n/%)	GIT (n/%)	Respiratory (n/%)	Yes (n/%)	No (n/%)	Don’t know (n/%)
Sex (*n = 61*)	Female	9/30	5/16.7	16/53.3	23/76.7	11/36.7	11/36.7	26/86.7	2/6.7	2/6.7
	Male	25/80.6	1/3.2	5/16.1	18/58.1	10/32.3	11/35.5	29/93.6	2/6.5	0
	Total	34/55.7	6/9.8	21/34.4	41/67.2	21/34.4	22/36.1	55/90.1	4/6.6	2/3.3
Age (*n = 43*)	18–25	6/80	0	4/40	7/70	4/40	3/30	7/70	2/20	1/10
	26–33	15/68.2	2/ 9.1	5/ 22.7	16/72.7	8/36.1	11/50	20/ 90.9	1/ 4.6	1/ 4.6
	34–41	1/20	1/20	3/ 33.3	4/80	1/20	1/20	5/100	0	0
	42–49	1/33.3	1/33.3	1/ 33.3	1/33.3	2/66.7	1/33.3	3/100	0	0
	> 49	2/66.7	1/33.3	0	2/66.7	0	1/33.3	3/100	0	0
	Total	25/58.1	5/ 11.6	13/30.2	30/69.8	15/ 34.9	17/ 39.5	38/88.4	3/7	2/4.7
Education (*n = 60*)	Diploma	19/51.4	4/10.8	14/37.8	24/64.7	9/24.3	12/32.4	35/94.6	1/2.7	1/2.7
	Degree	12/60	2/10	6/30	15/75	9/45	8/40	16/80	3/15	1/5
	MSc	3/100	0	0	2/66.7	3/100	2/66.7	3/100	0	0
	Total	34/56.7	6/10	20/33.3	41/68.3	21/35	22/36.7	54/90	4/6.7	2/3.3
District (*n = 62*)	Adigrat	8/32	5/20	12/48	18/72	6/24	6/24	19/76	4/16	2/8
	Gulomkada	15/88.2	0	2/17.8	10/58.8	10/58.8	8/47.1	17/100	0	0
	Ganta-afeshum	12/60	1/5	7/35	14/70	5/25	8/40	20/100	0	0
	Total	35/56.5	6/9.7	21/33.9	42/67.7	21/33.9	22/35.5	56/90.3	4/6.5	2/3.2
Profession (*n = 62*)	HHPs	22/44.9	6/12	21/42.9	34/69.4	17/34.7	16/32.6	43/87.8	4/8.1	2/4.1
	AHEs	13/100	0	0	8/61.5	4/30.8	6/46.1	13/100	0	0
	Total	35/56.5	6/9.7	21/33.9	42/67.7	21/33.9	22/35.5	56/90.3	4/6.5	2/3.2

^a^there were individuals who knew more than two form of the disease

^b^Leishmania, fly

#### Knowledge of symptoms, transmission, and control/prevention methods in animals

##### Community members

The number of community member respondents who knew one or more symptoms, transmission, or control/prevention methods of anthrax in animals was 26.3 (210/800), 21.3 (170/800) and 43.4% (347/800), respectively. Respondents who did not know symptoms, transmission, or control/prevention methods of anthrax were 73.8 (590/800), 78.8 (630/800) and 56.6% (453/800), respectively. The most well known symptom, transmission route, and control/prevention methods by the communities were sudden death in cattle (14.4%), ingestion grass contaminated by blood (13%) and isolation of anthrax infected animals (7.6%), respectively (Table 4).

**Table 4:** knowledge of the community members and professional respondents towards anthrax symptoms, transmission routes and control/prevention methods in animals

	Professionals		Community	
Variable	Frequency (***n = 62***)	%	Frequency (***n = 800***)	%
Number who did not knew anthrax symptoms (n/%)	16	25.8	590	73.8
Number who knew anthrax symptoms (n/%)	46	74.2	210	26.3
Sudden death	33	53.2	115	14.4
Un-clotted dark red blood	26	41.9	32	3
Bleeding from natural orifices	29	46.8	24	4
Incomplete rigor mortis	17	27.4	14	1.8
Other (swelling, wound, pain, fatigue, etc)	8^a^	12.9^a^	78	9.8
Number who did not knew anthrax transmission (n/%)	13	21	630	78.8
Number who knew anthrax transmission (n/%)	49	79	170	21.3
Ingesting of blood contaminated grass	33	53.2	104	13
Drinking contaminated water	30	48.4	76	9.5
Licking anthrax dead bones	24	38.7	33	4.1
Through contaminated soil	35	56.5	67	8.4
Believe its transmission but did not tell the method	7	11.3		
Other (inhalation, contact)	–	–	7	0.9
Number who did not knew anthrax control/prevention methods (n/%)	13	21	453	56.6
Number who knew anthrax control/prevention methods (n/%)	49	79	347	43.4
Isolate/separate anthrax infected animals	33	53.2	61	7.6
Avoid with anthrax infected people	–	–	37	4.6
Burn all suspected anthrax animal carcasses	27	43.6	33	4.1
Bury all suspected anthrax carcasses	32	51.6	22	2.8
Vaccinate animals	42	67.7	1	0.1
Bury and burn all suspected anthrax carcasses	22	35.5	1	0.1
Using Traditional medicine	–		36	4.5

^a^swelling, black wound, diarrhoea, bloat

##### Professionals

The number of professional respondents who reported one or more symptoms, transmission or control/prevention methods of animal anthrax was 74.2 (46/62), 79 (49/62) and 80.7% (50/62), respectively. Sudden death (53.2%) and contaminated soil (56.5%) were the most common symptoms and transmission routes, respectively, while they reported that vaccination (67.7%) of animals was the most effective control/prevention method (Table 4).

#### Knowledge of symptoms, transmission, and control/prevention methods in humans

##### Community members

The number of respondents who knew at least one anthrax symptom in humans was 36.8% (294/800) which was greater than in animals (26.3%, 210/800), and fever (22.4%) was the most recognized human symptom. Moreover, respondents who mentioned one or more transmission route and control/prevention method of human anthrax were 20.1 (161/800) and 45.6% (365/800), respectively. The respondents stated that consumption of infected animal products (raw meat & milk) was the most common transmission route (15.9%) while vaccination of animals (34.5%) was an effective mechanism of control/prevention (Table 5).

**Table 5:** knowledge of the community member and professional respondents towards anthrax symptoms, transmission routes and control/prevention methods in humans

	Professionals		Community	
Variable	Frequency (***n = 62***)	%	Frequency (***n = 800***)	%
Number who did not knew anthrax symptoms (n/%)	6	9.7	506	63.3
Number who knew anthrax symptoms (n/%)	56	90.3	294	36.8
Fever	39	62.9	181	22.4
Chills	28	45.1	80	10
Fatigue	31	50	37	4.6
Skin rash/wounds	41	66.1	68	8.5
Coughing	22	35.5	16	2
Lack of appetite	29	45.2	25	3.1
Headache	25	40.3	15	1.9
Irritability	15	24.1	10	1.3
Diarrhea	17	27.4	7	0.8
Vomiting	21	33.9	8	1
Sweating	21	33.9	12	1.5
Other (swelling, wound, pain, itching, etc)	1^a^	1.6^a^	102	12.8
Number who did not knew anthrax transmission (n/%)	6	9.7	639	79.9
Number who knew anthrax transmission (n/%)	56	90.3	161	20.1
Eating infected animal product (raw meat & milk)	52	83.9	127	15.9
Handling infected animals and animal products without protective clothing	28	45.1	66	8.3
Through contaminated soil	24	38.7	28	3.5
Number who did not knew anthrax control/prevention methods (n/%)	5	8.1	435	54.4
Number who knew anthrax control/prevention methods (n/%)	57	91.9	365	45.6
Avoid contact with anthrax infected animals	32	51.6	59	7.4
Avoid contact with anthrax infected people	22	35.5	35	4.4
By avoiding eating anthrax infected animal products	44	71	63	7.9
Bury all suspected anthrax carcasses	34	54.8	28	3.5
Burn all suspected anthrax animal carcasses	28	45.1	20	2.5
Bury and burn all suspected anthrax carcasses	22	35.5	7	0.9
Vaccinate animals	42	67.7	276	34.5
Using traditional medicine	–	–	10	1.3
Other (Keeping good hygiene/sanitation and nutrition)	–	–	9	1.1

^a^itching

##### Professionals

The majority of professional respondents could name one or more anthrax symptoms (90.3%, 56/62), transmission routes (90.3%, 56/62), and prevention methods (91.9%, 57/62). The most recognized symptoms, transmission routes, and control/prevention methods were skin rash (cutaneous wound) (66.1%, 41/62), consumption of infected animal products (raw meat and milk) (83.9%, 52/62) and vaccination of animals (67.7%, 42/62), respectively (Table 5).

#### Attitude and practice towards anthrax

Fifty-two percent (416/800) and 32.4% (259/800) of the questionnaire participants believed that vaccination of animals could prevent anthrax in animals and humans, respectively. But although 4% (32/800) said that they had anthrax (*Megerem*) infected animals, about 28% (9/32) of them used traditional medication for their animals. Moreover, of the 10.5% (84/800) respondents who had an anthrax infected family member at some point, 71.4% (60/84) had visited local healers (Table 6).

**Table 6:** Attitude and practice of community member respondents

Question	Response	Frequency	%
What animal husbandry do you practice? (*n = 452*)	Free grazing	84	19
	Zero grazing	354	80.1
	Mixed (free and zero)	4	0.9
	No report	10	2
Have you ever had anthrax infected animal(s)? (*n = 800*)	Yes	32	4
	No	392	49
	Do not know	376	47
If yes, what action did you take? (*n = 32*)	Reported to the Veterinarian	8	25
	If died, buried the dead animal	5	15.6
	Consumed meat of the dead animal	0	0
	Remove away the dead animal	7	21.9
	Use Traditional medication (local medicine, bleeding and branded)	9	28.1
	No report	3	9.4
Has any member of your family infected with anthrax? (*n = 800*)	Yes	84	10.5
	No	404	50.5
	Do not know	312	39
If yes, what action did you take? (*n = 84*)	Took the person to health facility	3	3.6
	bought drug from pharmacy	0	0
	Took to a local healer	60	71.4
	We did nothing	2	2.4
	use both (took to health center and local healer)	2	2.4
	Do not know	1	1.2
	No report	16	19.1
Do you think that vaccination of animals can prevent anthrax in animals? (*n = 800*)	Yes	416	52
	No	44	5.5
	Do not know	340	42.5
Do you think that vaccination of animals can prevent anthrax in humans? (*n = 800*)	Yes	259	32.4
	No	134	16.8
	Do not know	407	50.9
Do you think that anthrax is a serious disease of animals? (*n = 800*)	Yes	78	9.8
	No	418	52.3
	Do not know	304	38
Do you think that anthrax is a serious disease of humans? (*n = 800*)	Yes	90	11.3
	No	406	50.8
	Do not know	304	38
Were your animals vaccinated against anthrax? (*n = 800*)	Yes	35	4.4
	No	28	3.5
	Do not know	737	92.1

### Qualitative

#### Knowledge of anthrax/*Megerem*

##### Geographic variation on local names for anthrax

The researchers approached the participants by using the local name *Megerem* which is commonly known in Tigray. However, the researchers gave the participants an opportunity to give the local name of the disease in instances where they failed to recognize the name *Megerem*. In these instances, researchers described the clinical signs of the disease in animals and humans and asked respondents what they called this disease. Farmers usually named diseases based on their clinical signs and the lesions/pathology they identified in the animals.

“I do not know the disease *Megerem*, but I have heard about it. But I know *Lalish* and *Gulbus* in animals” (male participant, Sebeya). This participant called another person for help. The second participant gave a similar opinion with some clarifications.

“I do not know *Megerem* in animals. Perhaps we can learn from you. But in cattle, there are other diseases I know. One is *Lalish*, enlargement of the spleen. Moreover, there is another fatal disease called *Gulbus* showing cramp like symptoms and shivering. This disease may sometimes be confined around the head (neck swelling), in this case, an animal may not die soon” (the second male participant from Sebeya). This idea was supported by most of the participants in this FGD (Sebeya). The FGD participants from Ganta-afeshum district had a similar understanding about the disease. A 78 year-old male participant named the disease in animals *Tafia* (enlargement of the spleen) (Hagereselam tabia, Ganta-afeshum district). Similar suggestions had been given in Bizet tabia of the same district. Notably, a female participant told that *Lalish* was a severe disease of cattle (Bizet tabia, Ganta-afeshum district).

##### Anthrax/*Megerem* perceived only as human disease

The second participant from Sebeya said that *Megerem* appears in the neck/face of humans and could not be cured without treatment (modern medication). Another female participant from the same area shared her knowledge about human *Megerem*. “My daughter (one year old) was sick (swelling in the wrist). My daughter was waiting without medication for a few days. When the swelling had become bigger and bigger, I took my daughter to the health center. After medication, she was cured but she suffered.”

The researcher asked: “What does the swelling look like?” The same female participant responded: “the swelling starts out small. Then it increases in size with a depressed black eschar in the center.”

The researcher asked another question: “Do you think that this disease can affect animals?” “I do not know” she said. “Where did your daughter acquire the disease?” “I did not know its origin or where it came from. If I had known that I could have prevented and/or taken quick measures for my daughter during her suffering.”

Indeed, the second female participant from the same group partially supported the idea of the first female participant but she had a different view of the characteristics of the disease. This participant did not agree with the first female participant, especially with the nature of the lesion (depressed black eschar) in the center of the swelling. She said that the swelling had no depressed black eschar (Sebeya tabia, Gulomkada district). A female participant from Bizet tabia emphatically said that they should not be talking about *Lalish* in front of animals because animals could be panicked when they heard the word *Lalish*; indeed, she reflected the belief of the community. However, she failed to relate this to human *Megerem* (Bizet tabia, Ganta-afeshum district).

##### Anthrax perceived by the participants after they had been told its clinical signs

Most FGD participants from Fatsi and Anbesetefikada tabias (both from Gulomkada district) stated that they did not know the disease anthrax/*Megerem*, and that the disease has not occurred in their area. However, after the researchers had explained the nature/signs of the disease in animals and humans, a few individuals tried to share what they have heard/known about the disease.

“I have seen bleeding from natural orifices of dead animals. But I do not know the name of the disease” (male participant, Fatsi). Other participants said that they have seen bleeding through natural orifices and absence of rigor mortis of dead animals but they did not relate these signs to *Megerem* (Hagereselam and Bizet, Ganta-afeshum district).

#### Knowledge of the causative agent, transmission, and control/prevention methods for anthrax/*Megerem*

Most FGD participants did not know the causative agent, transmission, or control/prevention methods for anthrax/*Megerem* in animals and humans. Some of the participants associated *Megerem* in animals with the local belief Weqh’e (unidentified cause, but they stated that it caused sudden death), but the participants believed that it can be transmitted to humans through consumption of meat. Some of the participants also believed that the disease could occur in humans when there was stress (e.g. thirst, starvation), and consumption of meat (in humans), and alcohol (in humans and animals) might exacerbate the disease. According to these participants, the disease was commonly seen in animals with poor body condition and exacerbated when diseased animals had consumed water. Few participants mentioned that the disease was caused by microbes/germs. Regarding the control/prevention methods, FGD participants agreed that although the disease had been treated using traditional medicine, nowadays modern medication has become their best option in animals and humans. However, some of the participants still believed in traditional medicine, including heating the spleen using a hot iron or, if there was swelling around the bottom part of the neck. Moreover, bloodletting in animals were commonly used. None of the FGD participants recognized the GI and pulmonary forms of the disease in humans.

#### Attitude and practice

Some of the participants knew of the disease (in animals) by other names, including *Lalish*, *Tafia* (splenomegaly) and *Gulbus* (abdominal cramp and shivering). Some had perceived the disease only as a human disease while others recognized the animal disease after its clinical signs in animals and humans were described. Most of the participants did not know the transmission routes of the disease among animals, nor its zoonotic importance. However, during the discussion, they remembered precepts from the general health education given by the experts, e.g., not to eat the carcasses of dead animals. Some of the participants said that they were told not to eat animals that had died from anthrax/*Megerem*.

“I have heard that if animals died of *Megerem*, their meat should not be eaten and their blood should not be touched. But the community does not follow these recommendations” (male participant, Fatsi). In Sebeya, the researchers raised the issue of the outbreak that occurred in 2018 and asked the participants about the transmission routes and control/prevention measures that were taken: “We had been informed that a girl (tenth grade student) had died from eating dead animal meat. And experts told us that the animal died from a disease in which the carcass should not have been eaten *…*” (Participants from Sebeya).

According to the key informants, the eating habits and lifestyle of the communities are high risk for contracting anthrax. “Indeed, no animals have died from anthrax. But when animals die for any reason (for example rabies), individuals will resist burying the carcass; they need to consume it” (Meida Agame health center director, Adigrat town district). Other key informants said that the communities often live with their livestock in the same house, and they slaughter animals for consumption at home (i.e., they never use an abattoir) (Bizet health center director, Ganta-afeshum district). Another challenge reported by officers was the negative attitude of the community towards animal vaccination against infectious diseases (including anthrax) (Gulomkada district animal health and Adigrat Veterinary clinic coordinators). Instead, they prefer visiting the local healers over modern treatment and vaccination (Gulomkada District Health coordinator).

#### Reasons for low KAP of the community towards anthrax

In general, the low KAP towards anthrax can be attributed to three primary drivers:

1. **Animal and human health officers**

##### Anthrax as a forgotten disease

Key informants already admitted that their respective communities had low KAP towards anthrax because the prevention of the disease was not a priority in the study area:

“Our community has a low awareness of anthrax because we do not deliver adequate health education specific to anthrax. Since the disease is not common, it is not in our top list. However, we inform the community about the general impact of zoonotic diseases like tuberculosis and rabies” (Fatsi health center director). Likewise, the director of Tekli Siwuat health center of Adigrat said, “we have no scheduled separate prevention program against anthrax. But we try to associate it with our rabies prevention program” (according to the director, rabies outbreaks were common in the town).

Similar suggestions had been given by other human health officers (Adi-aynom and Meida Agame health center officers from Ganta-afeshum and Adigrat town districts, respectively). They said that anthrax cases were not admitted to their health centers. Moreover, they stated that the disease had not occurred in their surroundings and that this was why health education given specifically for anthrax was poor. One of the key informants described anthrax as a “forgotten” disease. “A long time ago there were rare anthrax cases but nowadays it is being forgotten” (Meida Agame health center director, Adigrat town district).

##### Irregular health education

Some of the key informants said that health education was conducted in Sebeya and Bizet where anthrax outbreaks had occurred in their localities. The director of Sebeya health center said “there was an outbreak that killed a girl and left others infected in a village called Adibeteksian [found in Sebeya tabia]. The source of the case was a cow that died from anthrax. The girl was presented to our clinic with severe abdominal cramps and fever. The cause was ultimately diagnosed as anthrax (by Aider specialized hospital in Mekelle, the regional capital), but we failed to save the life of that innocent girl. Since that time, we have begun health education together with the veterinarians in the area. We started teaching the community during the funeral of the girl that it was because of the delay that she lost her life, and that they must immediately bring their children when they observe such signs and should avoid consuming animals that are sick and even the carcasses of apparently healthy animals which are not properly inspected.”

The animal science and health district coordinators of Gulomkada confirmed the above suggestions, and after the outbreak of anthrax occurred in Sebeya (in 2018), they created anthrax-oriented educational programs for Sebeya and its surroundings. However, during our interviews, we found that although health education was concentrated in areas where there were anthrax outbreaks, education was not continued once the outbreak had been controlled: “During the anthrax outbreak (to control the disease) we were cooperating with human health professionals in an integrated way. However, after the disease was under control we did not continue to work together for prevention purposes” (Animal science district coordinator in Gulomkada).

2) **Government**

Until recently, anthrax education and mitigation has not been a priority for the Ethiopian government. Funding specificied for anthrax prevention, education, and human vaccination has not been provided.

“To date, we have more than 15 vaccines in stock but there is no human anthrax vaccine, either independently or in combination with others, among the 15” (Fatsi health center director, Gulomkada district). Moreover, although anthrax can be prevented using ante- and postmortem inspections, the Bizet health center director in the Ganta-afeshum district reported that there was proper abattoir and slaughtering methods were not being utilized. The officer added that investors were not willing or able to invest in abattoirs. Other officers reported that there were inadequate veterinary clinics and drugs (Animal science district coordinator of Gulomkada) as well as man power (Adigrat veterinary clinic coordinator) to tackle the disease. “There are also remote areas that we cannot access it via vehicles even during anthrax epidemics. … Nowadays the community is reluctant to having people gather for health education; the community may need mass media communication that allows them to stay at home” (Gulomkada District Health coordinator).

3) **Community**

The negative attitude of the community towards anthrax contributed its own challenge to the prevention of anthrax. Focus Group Discussions and KIIs revealed that the community has continued to maintain local beliefs. Even though the communities have not been provided adequate education, they resist implementing what little they have been told, and restrict themselves to the traditional way of living.

## Discussion

Anthrax is a neglected tropical disease, and it is seldom studied in Ethiopia. Pieracci et al. [28] have prioritized anthrax as the second most significant zoonotic disease in Ethiopia based on its negative impacts at the household level due to causing disease and production loss in livestock, as well as severe disease in human. This study confirms that anthrax had not yet been appropriately prioritized in Ethiopia. Key informants from Gulomkada reported that anthrax had been a problem in their community, having caused (human) death and socio-economic crisis. Another key informant from Adigrat said that anthrax was a forgotten disease. This indicates that there was no anthrax control/prevention program coordinated by the central government. In our study, socio-demographic/social differences of the study community (age, sex, animal ownership, and district) were significantly associated with the knowledge of the disease.

Link and Phelan [35] indicate that social conditions (e.g. race, socioeconomic status, gender, and other stressful life events of a social nature) are associated with fundamental causes of disease. Moreover, In West Africa, during the Ebola epidemic, the traditions of local communities frequently caused challenges in terms of controlling the disease [36]. These problems could be mitigated, if not solved, by education. The Health Belief Model posits that messages will achieve optimal behavioral change if they successfully target perceived barriers, benefits, self-efficacy, and threat [37].

Even though 62% of the respondents stated that they knew the disease anthrax/*Megerem*, only 9.3% of them explained its causative agent, i.e. the microbe/germ. About 9 and 4.3% believed that the disease was God-given and caused by other entities (heredity, witchcraft, lack of sanitation, hunger, insects, thirst), respectively while 77.6% did not know the causative agent of the disease. Among the listed clinical signs, only 26.3 and 36.8% of the respondent could name at least one sign in animals and humans, respectively. During the questionnaire survey data collection, we observed that some respondents perceived staphylococcal skin infections, locally (in *Tigrigna*) called *Migli Chiwa*, as cutaneous anthrax in humans. We also encountered a similar condition in animals. According the FGDs, the swelling which developed around the lower neck of animals (locally called *Zigag*) was not fatal. This might be mistaken with the throat swelling caused by subacute form of anthrax [1]. Qualitative results demonstrate a poor understanding of the disease overall among the study community. In fact, similar findings were reported from Zambia: quantitative results showed good awareness among respondents while qualitative results indicated poor knowledge concerning the disease in the same communities [27].

More than 38% of the respondents had no formal education. It was also reported by the key informants that except in Sebeya (where education was provided during an outbreak), health education was not provided. Hence, it was not expected that the study community could have been aware of the disease. This scenario is similar with Sitali et al. [27] who suggested that education influences one’s access to information and ability to comprehend health messages. Even involvement of an educated family members in farming practices can create awareness and improve knowledge about zoonotic disease [31]. Indeed, the present study community was not adequately exposed to public health messages. Among the survey respondents who claimed to have known anthrax (496/800), about 90% (446/496) of them acquired information regarding the disease from family, friends and neighbours/colleagues, which increases the likelihood that community members are exposed to misconceptions and myths surrounding the disease [27].

During the FGDs, some participants said that they did not know *Megerem* in animals but *Lalish* and *Tafia* (splenomegaly) and *Gulbus* (abdominal cramp and shivering), and others described clinical signs like bleeding in dead animals. These circumstances indicate the absence/lack of consistent health education in the study area. Consistent with this study, Opare et al. [25] showed that most respondents do not know the causes of anthrax but recognize the signs of the disease. Moreover, in the questionnaire survey, the number of respondents who knew the clinical signs was higher than that of respondents who knew the cause of the disease. Although 74.2 and 90.3% of the professional respondents could name at least one sign of the disease, only 55.7% of them knew the causative agent of the disease.

About 10 and 11% of the respondents felt that anthrax was a serious disease of animals and humans, respectively, and 52 and 32.4% of them believed that vaccination could prevent the disease in animals and humans. However, only 4.4% (35/800) of them have vaccinated their animals. In general regarding the KAP of the respondents on anthrax, we observed that knowledge was better than attitude, and attitude was better than practice. This is supported by a study conducted in Ghana which indicated that high levels of knowledge of the farmers on vaccination had not been realized as practices [25].

In fact, practice might be influenced by culture and socio-economic factors. The deep-rooted belief could not be changed unless the Health Belief model variables are successfully inculcated in the community. This model suggested that individuals who perceive a risk which can cause low health problems are unlikely to engage in behaviors to reduce their risk of developing that particular health problem; hence, optimal behavioral change is achieved if the Health Belief Model successfully target perceived barriers, benefits, self-efficacy, and threat [37].

Participants from Sebeya tabia said that they had been informed that the meat of an infected animal was the cause of a local girl’s death. However, the participant reported reluctance towards heeding the public health messages provided. During the recent Ebola outbreak in West Africa, some of the local people not only detached themselves from help but they also actively fought the prevention program delegations and killed eight members. The control and prevention program was finally successful when the community was made part of the program using social mobilization action [36]. Other reports indicate that how notions of ‘the community’ can be problematic if used uncritically [38, 39].

On the other hand, socio-economic factors could affect a practice made to avoid a given risk. One of the key informants pointed out “indeed poverty is our main challenge”. This followed the claim that the community had lived with their livestock in the same house. According to other key informants, the community resisted burying the carcasses of dead animals. Consumption and selling of carcasses in which the animals died from anthrax was reported by other studies; this is not only to make financial return but also as a source of protein [14, 25, 27, 40]. Key informants also said that there were remote and inaccessible areas which could not obtain veterinary services. Sitali et al. [13, 27] report that practices that can be used to prevent anthrax have been impacted by infrastructure. Bruce and Phelan (1995) postulate that the essential feature of fundamental social causes involves access to resources (e.g., money, knowledge, power, prestige) that can be used to avoid risks or to minimize the consequences of disease once it occurs. Likewise, a similar theory was formulated by Phelan et al. [41] which states that differences in socio-economic status bring inequality in health.

This study was limited in several ways. For example, there was no common local name for anthrax in the study areas. The local name, *Megerem*, is widely known in Tigray. But some of our study village residents referred to the disease by different local names. During the interviews, the respondents did not recognize the disease (their response might be “No” or “I do not know”), or they understood the disease differently (mostly *Megli Chiwa* in human and *Zigag* in animals - both are non-fatal swellings). Having identified this problem, we trained our interviewers on how to approach the respondents who had different understandings of the disease. The presence of local veterinarians during the discussion might also have biased the response of the FGD participants.

## Conclusion

In general, the KAP of the participants towards anthrax was low. Moreover, there was no consistent understanding of the disease among the participants. The study also revealed that the participants did not get consistent, adequate, and continuous health messages regarding the disease. Traditional belief and socio-economic factors impacted the KAP of the community towards the disease. Hence, community attitudes towards health-related behaviors were frequently adversely affected by traditional approaches towards medical care (both in veterinary and human). It is the responsibility of the government and the experts in improving this tradition. This can effectively be done through community-centered programs.

## Supplementary Information

**Additional file 1.**

## Data Availability

Data will not be made public to keep the confidentiality of the participants but can be obtained from authors on reasonable request.

## Abbreviations

AHEs: Animal Health Experts
AHOs: Animal Health Officers
*B. anthracis*: *Bacillus anthracis*
CI: Confidence Interval
FGD: Focus Group Discussion
GI: Gastro Intestinal
HHOs: Human Health Officers
HHPs: Human Health Practitioners
KAPs: Knowledge, Attitude and Practices
KII: Key Informant Interview
OR: Odds Ratio
US-CDC: United States Centers of Disease Control
