# Supplementary material for: Knowledge, attitude and practice towards anthrax in northern Ethiopia: a mixed approach study
Source: BMC Infect Dis. 2020 Nov 10;20:814. doi: 10.1186/s12879-020-05544-z (PMC7653774; doi:10.1186/s12879-020-05544-z)
Supplement: Supplementary file 1 — Additional file 1. [file 12879_2020_5544_MOESM1_ESM.docx]

No:________

**Questionnaire A: Questions for community members**

This study aims to collect information on knowledge, attitudes and practices (KAPs) regarding anthrax among community members living in Ganta-afeshum, Golomekeda and Adigrat town districts. You are being asked to participate in this study as a community member from this area and would be grateful if you are willing to participate by answering questions from this questionnaire. I assure you that all the information collected from you will be kept confidential. You may refuse to answer any particular question and may stop the interview at any time.

Do you agree to participate and answer questions in this study? A) Yes B) No

Name of Enumerator____________________Tel. No. _____________

**I) PERSONAL INFORMATION**:

1. Name of participant: ______________________Tel. No. _______________

District:_____________ Tabia: ___________ Kushet/Ketena: __________Date of interview: dd/mm/yyyy ______________

**II) SOCIO-DEMOGRAPHIC INFORMATION**:

2. Age (years) _________

3. Gender: A) Male B) Female

4. Level of education

A) No formal education

B) Primary – 1-4

C) Junior - 5-8

D) secondary school – 9-10

E) Preparatory - 9 – 12

F) Diploma

G) Degree

H) MSc and above

5. Occupation

A) Government employed B) Farmer C) Merchandiser D) Student E) daily worker

F) Other (specify) ____________________________________________________

6. Religion

A) Christian B) Muslim C) Catholic D) Other (specify) ________________________

**III) ANIMAL OWNERSHIP**

7. Do you have animals? A) Yes B) No

8. If yes, please put the number, and indicate for what purpose you are using

| Animal type | No. Female | No. Male | Total No. | Purpose |
| --- | --- | --- | --- | --- |
| Cattle |  |  |  |  |
| Goats |  |  |  |  |
| Sheep |  |  |  |  |
| Donkeys |  |  |  |  |
| Horse |  |  |  |  |
| Camel |  |  |  |  |
| Other (Specify) |  |  |  |  |

**IV) KNOWLEDGE AND AWARENESS TOWARDS ANTHRAX**

9. Do you know a disease called anthrax?

A) Yes B) No

10. If yes, where did you learn about it? (Check all mentioned)

A) Newspapers and magazines

B) Radio

C) TV

D) Veterinary officials

E-Brochures, posters and books

F) Medical officials

G) Teachers

H) Religious leaders

I) Family, friends, neighbors/colleagues J) Other (Specify)_________________________

11. What do you think about the cause of the disease?

1. Germs B) God given

C) Don’t know D) other (Specify) ______

12. Does anthrax transmit among humans? A) Yes B) No C) Do not know

13. If yes how? ________________________________________________________________

14. Does anthrax transmit from humans to animals? A) Yes B) No C) Do not know

15. If yes, how? ________________________________________________________________

16. Does anthrax transmit among animals? A) Yes B) No C) Do not know

17. If the answer is yes how?

A) By ingesting blood contaminated grass

B) By drinking contaminated water

C) By licking anthrax dead bones

D) Through contaminated soil

E) Do not know

F) other (specify)__________

18. Does anthrax transmit form animals to humans? A) Yes B) No C) Do not know

19. If yes how is anthrax transmitted to humans?

A) Eating infected animal product

B) By handling infected animals during skinning without protective clothing

C) Through contaminated soil

D) Do not know E) other (specify)____________________

20. Have you ever seen a person with anthrax? A) Yes B) No

21. If yes, where?

A) Through media (TV) B) Personal observation C) Other (specify____________________

22. How can we prevent animals from getting anthrax? (Select all that apply)

A) Isolate/separate anthrax infected animals

B) Prevent contact between animal and anthrax infected people

C) Burn all suspected anthrax animal carcasses

D) Bury all suspected anthrax carcasses

E) Vaccinate animals

F) Bury and burn all suspected anthrax carcasses

G) Using Traditional medicine

H) Do not know

I) other (specify) _______________________________________

23. How can a person prevent him/herself from getting anthrax? (Select all that apply)

A) Avoid contact with anthrax infected animals

B) Avoid contact with anthrax infected people

C) By avoiding eating anthrax infected animal products

D) Bury all suspected anthrax carcasses

E) Burn all suspected anthrax animal carcasses

F) Bury and burn all suspected anthrax carcasses

G) Vaccinate animals

H) Using traditional medicine

I) Do not know

J) other (specify)_________________

24. What are the clinical signs/symptoms of an animal with anthrax? (Select all that apply)

A) Sudden death B) Bleeding from natural orifices

C) Unclotted dark red blood D) Incomplete rigor mortis

E) Do not know F) Other (specify)______________________________

25. Have you ever had anthrax Patient Animals? A) Yes B) No

26. If Yes, Please Fill the table below, accordingly

| Animal type | Female | | | | Male | | | |
| --- | --- | --- | --- | --- | --- | --- | --- | --- |
|  | Age | Before This year | This year | Disease outcome | Age | Before this year | this year | Disease outcome |
| Cattle |  |  |  |  |  |  |  |  |
| Goats |  |  |  |  |  |  |  |  |
| Sheep |  |  |  |  |  |  |  |  |
| Donkeys |  |  |  |  |  |  |  |  |
| Horse |  |  |  |  |  |  |  |  |
| Camel |  |  |  |  |  |  |  |  |
| Other (Specify) |  |  |  |  |  |  |  |  |

27. What are the clinical signs/symptoms of a person with anthrax? (Check all that apply).

A) Fever B) Chills C) Fatigue (extreme tiredness) D) Skin rash/wounds E) Coughing F) Lack of appetite G) Headache H) Irritability I) Diarrhea

J) Vomiting K) Excessive sweating

**V) ATTITUDE AND PRACTICE**

28. Do you think that anthrax is a serious disease in humans and animals in your locality?

I) Human

A) Yes B) No c) do not know

II) Animal

A) Yes B) No c) do not know

29. What animal husbandry do you practice?

A) Zero grazing C) Mixed - free range and zero grazing

B) Free range D) Other (Specify) _______________________________________

30. Where do you get fodder for your animals?

A) Graze in the field C) Buys commercial fodder

B) Cut and carry fodder D) Other (Specify)________________________________

31. Have you ever had anthrax infected animal(s)?

A) Yes B) No C) Don’t know

32. If yes for question No 31, what actions did you take?

A) Reported to the Veterinarian B) If died, buried the dead animal without reporting

C) Consumed meat of the dead animal D) Remove away the dead animal

E) Other (Specify) _____________________

33. Has any member of your family infected with anthrax?

A) Yes B) No C) Don’t know

34. If yes for question No 33, how did the person contract it?

A) Skinning dead animal C) Carrying hide form dead animal

B) Eating dead animal D) while carrying meat from dead animal

E- Other (Specify) ______________________________________

35. From the above question; what action did you take?

A) Took the person to the nearest health facility

B) We bought drug from pharmacy

C) Took the person to a traditional healer

D) We did nothing

E) Other (Specify) ________________________________________________________

36. Has anthrax outbreak occurred in your area? A) Yes B) No C) Don not know

37. If yes for question No 36, when?

A) Within this 6months ago B) Within this year

C) one year ago D) Don not Know E) Other (Specify) ________________

38. Was there vaccination during the period of outbreak? A) Yes B) No C) don’t know

39. Were your animals vaccinated against anthrax? A) Yes B) No C) Do not know

40. If No which animals were not vaccinated and why? ________________________

41. How often is animal vaccination against anthrax done in your area?

A) Twice a year B) Once a year

D) Never Vaccinated E) Other (specify)___________________________

42. What prompts you to take your animals for vaccination?

A) To protect animals C) because others do so

B) To protect humans D) we are forced by government officials to do so

E) Other (Specify)_______________________________________________

43. If you do not always take your animals for vaccination, what are the reasons?

A) No Veterinary services C) The vaccination center is far

B) Financial problems D) Don’t get informed when it occurs

E) Other (Specify)______________________________________________________

44. Do you think that vaccination of animals can help to prevent anthrax in animals?

A) Yes B) No

45. Do you think that vaccination of animals can help to prevent anthrax in humans?

A) Yes B) No

No:________

**Questionnaire B: Questions for health professionals**

This study aims to collect information on knowledge, attitudes and practices (KAPs) regarding anthrax among community members living in Ganta-afeshum, Golomekeda and Adigrat town districts. You are being asked to participate in this study as a Health professional and would be grateful if you are willing to participate by answering questions from this questionnaire. I assure you that all the information collected from you will be kept confidential. You may refuse to answer any particular question and may stop the interview at any time.

Do you agree to participate and answer questions in this study? A) Yes B) No

Name of Enumerator_________________________________________

Telephone number of enumerator_______________________________

I) **PERSONAL INFORMATION**

Name of participant: ___________________________________

Telephone number of participant (optional): ________________________

District: ________________ Tabia: ______________________

Kushet: __________________ Date of interview: dd/mm/yyyy ______________

**II) SOCIO-DEMOGRAPHIC INFORMATION**

Age (years) _________

Gender: A) Male B) Female

1. Level of education

A) Diploma

B) Degree

C) MSc

F) PhD

2. Religion

A) Christian B) Muslim C) Catholic D) Other (specify) ________________________

**III) ANTHRAX KNOWLEDGE AND AWARENESS**

5. What is the Etiology of anthrax? _____________________________________

6. What are the forms of anthrax in humans? A) Cutaneous B) Gastrointestinal C) Respiratory D) Other (mention)__________________________________________

7. What are the transmission routes of anthrax in humans for the respective form listed below?

A) Cutaneous___________________________________________________________

B) Gastrointestinal _______________________________________________________

C) Respiratory ______________________________________________________

D) Other (mention) ______________________________________________________

8. Does anthrax transmit among humans? A) Yes B) No

9. If yes how?__________________________________________________________________

10. Does anthrax transmit among animals? A) Yes B) No

11. If the answer is yes how?

A) By ingesting blood contaminated grass

B) By drinking blood contaminated water

C) By leaking anthrax dead bones

D) Through contaminated soil

E) Do not know

12. Does anthrax transmitted form animals to humans? A) Yes B) No

13. If yes how is anthrax transmitted to humans?

A) Eating infected animal product

B) By handling infected animals without protective clothing

C) Through contaminated soil

D) Do not know

14. Have you ever seen a person with anthrax? A) Yes B) No

15. If yes, where?

A) Through media (TV)

B) Personal observation

C) Other (specify) _________________________________________________

16. How can we prevents from get infected with anthrax?

A) Avoid anthrax infected animals

B) Burn all suspected anthrax animal carcasses

C) Bury all suspected anthrax carcasses

D) Vaccinate animals annually

E) Do not know

17. How can a person prevent him/herself from getting anthrax? (Select all that apply)

A) Avoid anthrax infected animals

B) Avoid anthrax infected people

C) Burn all suspected anthrax animal carcasses

D) Bury all suspected anthrax carcasses

E) Avoid skinning anthrax dead cadaver

F) Vaccinate animals

G) Do not know

18. What are the clinical signs/symptoms of an animal with anthrax? (Select all that apply)

A) Sudden death

B) Bleeding from natural orifices

C) Un clotted dark red blood

D) Incomplete rigor mortis

E) Other (specify)_______________________________________________________

19. What are the clinical signs/symptoms of a person with anthrax? (check all that apply).

A) Fever

B) Chills

C) Fatigue (extreme tiredness)

D) Skin rash/wounds

E) Coughing

F) Lack of appetite

G) Headache

H) Irritability

I) Diarrhea

J) Vomiting

K) Excessive sweating

**IV) ATTITUDE AND PRACTICE**

20. Do you think that anthrax is a serious disease in humans and animals in your locality?

I) Human

A) Yes B) No c) do not know

II) Animal

A) Yes B) No c) do not know

21. Have you had anthrax outbreak in the area? A) Yes B) No

22. If yes, when?

A) Less than 6months ago B) More than 1year ago

A) 1year ago B) Other (Specify) …....................

23. Do you think that vaccination of animals can help to prevent anthrax in animals?

A) Yes B) No

24. Do you think that vaccination of animals can help to prevent anthrax in humans?

A) Yes B) No

**Questionnaire C: Thematic question of FGD and KII**

**I. Themes of focus group discussion (FGD)**

1. Do you know the disease anthrax? Or have you ever heard about the disease anthrax?

2. What is the cause of the disease?

3.Do you know the signs of anthrax in animals and humans?

4.What are the transmission methods? How humans do acquire the disease? How animals do acquire the disease?

5. What are the control/prevention methods of the disease?

**II. Themes of Key informant interview (KII)**

1. Anthrax case (animal/human) ever admitted in your clinic/hospital?
2. Has anthrax outbreak ever occurred in this locality?
3. What are the control/prevention measures which are being taken?
4. What the challenges that are facing during the application of control/prevention methods?

**Fig A: Knowledge of respondents on anthrax based on age**

**Table A: The methods of acquiring information about anthrax?** (n=800)

| Where did you learn about anthrax | Frequency | % |
| --- | --- | --- |
| Newspapers and magazines | 4 | 0.5 |
| Radio | 17 | 2.1 |
| Television | 7 | 0.9 |
| Veterinary experts | 16 | 2 |
| Brochures, posters and books | 2 | 0.3 |
| Health experts | 22 | 2.8 |
| Teachers | 6 | 0.8 |
| Religious | 0 | 0 |
| Family, friends, neighbors/colleagues | 446 | 55.8 |

**Fig B: The methods of acquiring information about anthrax**
